# A novel technique for lead sparing tricuspid valve replacement in the case of a transvenous ICD lead

**DOI:** 10.1002/ccr3.1648

**Published:** 2018-06-29

**Authors:** Christian Paech, Franziska Wagner, Bianca Karthe, Farhad Bakthiary, Roman Antonin Gebauer

**Affiliations:** ^1^ Department for Pediatric Cardiology University of Leipzig ‐ Heart Center Leipzig Germany; ^2^ Department for cardiac surgery HELIOS Heart Center Siegburg Siegburg Germany

**Keywords:** cardiac pacing, congenital heart disease, tricuspid valve surgery

## Abstract

Tricuspid valve (TV) surgery represents a complex consideration regarding lead management in patients with a transvenous ICD. The presented case shows favorable short‐term results after lead sparing TV replacement, leaving an ICD lead in a paravalvular position. The described technique can be used in challenging cases of TV replacement.

## 1 INTRODUCTION

Tricuspid valve (TV) surgery represents a complex consideration with regard to lead management in patients with a transvenous ICD. The case of an adult with congenital heart disease is presented. To spare the transvenous ICD lead during TV replacement, a novel technique was used, leaving the ICD lead in a paravalvular position. Technique and outcome of the procedure are reported.

Tricuspid valve replacement is a standard procedure in the case of TV dysfunction.^1^ A considerable percentage of affected patients also have an implanted cardiac pacemaker or implantable cardioverter defibrillator with leads passing the TV. In these cases, tricuspid valve replacement represents an especially complex issue as the PM or ICD leads usually have to be explanted before and reimplanted after the procedure. This represents a considerable additional risk for the patient and the newly implanted valve.

## 2 CASE

**Figure 1:**
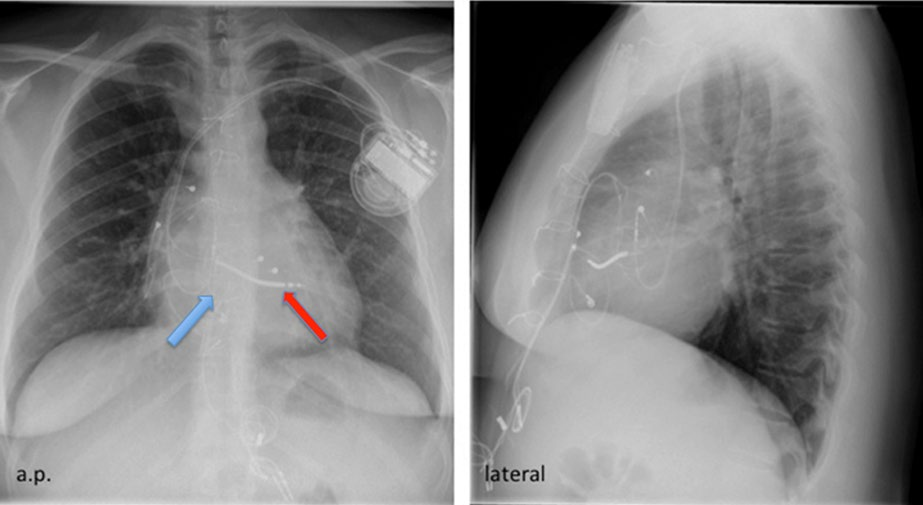
Preoperative chest X‐ray demonstrating the lead position before TV replacement. The red arrow marks the ICD lead, and the blue arrow marks the TV position

**Figure 2:**
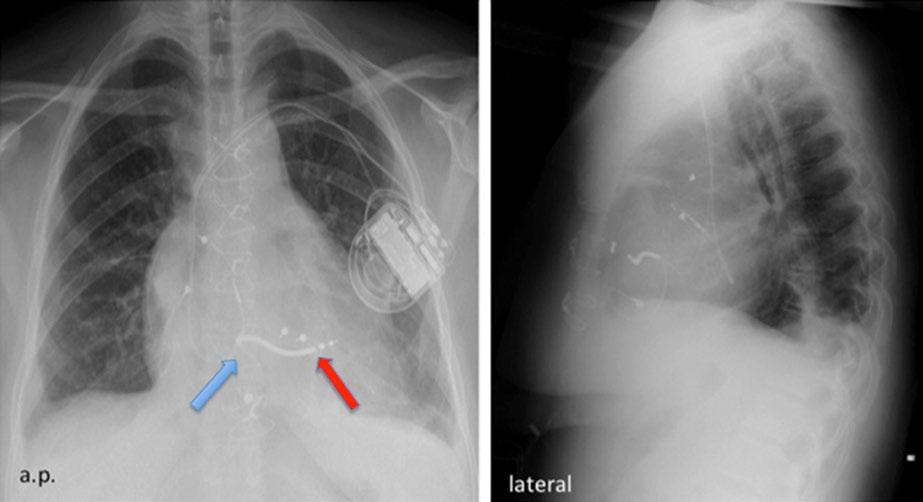
Postoperative chest X‐ray demonstrating the lead position after TV replacement. The red arrow marks the ICD lead, and the blue arrow marks the TV position

**Figure 3:**
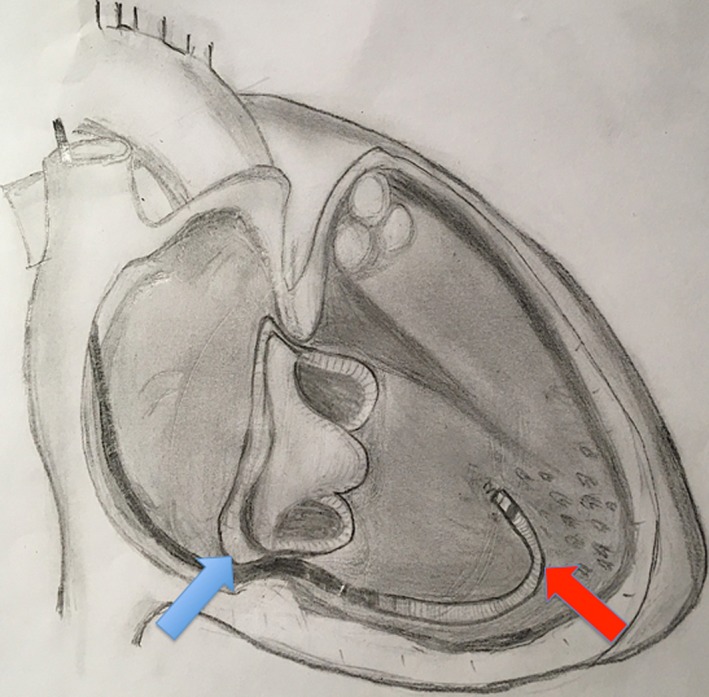
Schematic portrayal of the lead position after TV replacement. The red arrow marks the ICD lead, and the blue arrow marks the TV position

A 35‐year‐old female patient was presented to our center for TV replacement due to stenosis. At the age of 11 years, the patient suffered from endocarditis supported by a small ventricular septal defect. At that time, the ventricular septal defect was closed and a mechanical tricuspid valve (SJM, 28 mm) implantation and epicardial single chamber pacemaker implantation due to concomitant third degree AV block without escape rhythm were performed. At the age of 17 years, the abdominal pacemaker was replaced, due to battery depletion (Biotronik). Six months later, a second surgery followed with replacement of the dysfunctional mechanical valve for a 27 mm Porcin‐Baxter biological TV. Another PM replacement (Medtronic) was performed at the age of 27 years, again, due to battery depletion. At the age of 30 years, the abdominal pacemaker was explanted and a two chamber transvenous ICD (Medtronic Egida DR) was introduced, as the patient suffered a syncope during documented sustained ventricular tachycardia. The ICD lead was implanted passing the tricuspid valve into the right ventricle. Six years later, severe TV stenosis developed, caused by an ICD lead, that stuck to the posterior leaflet of the biological valve and ventricular myocardium, and the TV had to be replaced a third time. To avoid future TV dysfunction due to lead related complications, we discussed several options with the patient. These included first TV replacement in combination with epicardial pacemaker leads and a subcutaneous ICD (S‐ ICD, EMBLEM™ Boston Scientific) or alternatively placing a pacing lead in the coronary sinus in combination with an S‐ ICD. As the patient disagreed on both options, despite a higher risk of TV dysfunction by placing another transvenous ICD lead through the TV, we discussed another option and opted for a lead sparing replacement of the TV. The TV ring was cut open on both sides beneath the original ICD lead, and the TV prosthesis was excised. A new bioprosthetic valve was implanted, leaving the ICD lead outside the ring of the new TV prosthesis. As the lead was stuck to the former TV annulus and posterior ventricular wall, no further fixation of the lead was needed. The new valve (SJM biological 28 mm) was implanted using 14 Coreknots (Figures 1, 2, 3).

The postoperative course was unremarkable, and the patient was dismissed on the 11th postoperative day/postoperative day 11. Follow‐up after 4 months showed a well functioning TV prosthesis without stenosis or regurgitation and no deterioration in pacing or sensing parameters. No artifacts or other hints to a lead dysfunction were recorded (Table 1).

**Table 1:** Pre‐ and postoperative lead data

	Preoperative data		4‐month follow‐up after TV replacement	
	Atrium	Right ventricle	Atrium	Right ventricle
Amplitude (mV)	1.1	5,8	1.90	‐
Impedance (Ohm)	418	456	437	380
Amplitude of stimulation (V)	1.25	1.00	0.75	1.00
Pulse width (ms)	0.4	0.50	1.00	1.00

## 3 DISCUSSION

We present the first case of a lead sparing TV replacement, leaving an ICD lead in a paravalvular position.^1^

It is a common phenomenon that some patients, who need a TV replacement, need or already have an ICD or cardiac pacemaker. Several options come in mind when thinking about the type of device and implantation route. As has been initially performed in the presented case, there is the option of an epicardial pacemaker with the advantage of a nearly absent risk for endocarditis, harm to inner‐cardiac structures, and equal results for device longevity.^2^ Epicardial pacing is frequently used in congenital heart disease patients with the major disadvantage of an at least partial sternotomy that is needed to place or revise the pacing leads. An alternative approach is the placement of a pacing lead in the coronary sinus. A procedure has been published in case reports or small case series to be feasible.^3^ The subcutaneous ICD is the only alternative approach for patients, who exclusively need defibrillation instead of cardiac pacing.

Therefore, it is common practice to implant or reimplant a transvenous pacing or ICD lead, that passes the tricuspid valve prosthesis, in many patients after TV replacement. Major advantages are the easy implantation technique and a relatively easy removal in case of lead dysfunction. On the other hand, there are many reports on native, as well as bio‐prosthetic valve, complications in case of transvalvular implantation.^4^ In particular, TV stenosis, due to stuck valvular leaflets, perforation of a leaflet during implantation and papillary muscle perforation has been reported.^5^

With the lead sparing TV replacement technique used in the presented case, it can be speculated, that the risk of lead‐related valve dysfunction should be minimized and the risks associated with an exchange of the ICD lead completely avoided. A similar technique has been reported for PM leads by Shaikhrezai et al. and Yoshikai et al., who reported good short‐term results.^6^, ^7^ There are two major concerns about the technique presented. First of all, an elevated risk for lead dysfunction may be assumed, as the lead is fixed in a paravalvular position in relatively close contact to the ring of the valve prosthesis. Secondly, a removal of the lead in the future will be challenging, if not impossible, without damaging the TV. However, the later risk of a future lead dysfunction can be resolved by simply placing a new ICD lead in the traditional way passing the TV, leaving the paravalvular lead abandoned.

## 4 CONCLUSION

The presented case shows favorable short‐term results after lead sparing TV replacement, leaving an ICD lead in a paravalvular position. Yet, a longer follow‐up period is required to determine whether this technique is also reliable in the long term. The described technique can be used in challenging cases of TV replacement after thoughtful consideration of pros and cons.

## CONFLICT OF INTEREST

None declared.

## AUTHOR CONTRIBUTION

CP: drafted the manuscript. FW: assisted in drafting the manuscript. BK: critically reviewed the manuscript and performed data collection. FB: critically reviewed and approved the manuscript. RA: Gebauer critically reviewed and approved the manuscript.
